# Hemodilution on Cardiopulmonary Bypass as a Determinant of Early Postoperative Hyperlactatemia

**DOI:** 10.1371/journal.pone.0126939

**Published:** 2015-05-18

**Authors:** Marco Ranucci, Giovanni Carboni, Mauro Cotza, Paolo Bianchi, Umberto Di Dedda, Tommaso Aloisio, the Surgical and Clinical Outcome Research (SCORE) Group

**Affiliations:** Department of Cardiothoracic—Vascular Anesthesia and Intensive Care, IRCCS Policlinico San Donato, San Donato Milanese (Milan), Italy; Azienda Ospedaliero-Universitaria Careggi, ITALY

## Abstract

**Objective:**

The nadir hematocrit (HCT) on cardiopulmonary bypass (CPB) is a recognized independent risk factor for major morbidity and mortality in cardiac surgery. The main interpretation is that low levels of HCT on CPB result in a poor oxygen delivery and dysoxia of end organs. Hyperlactatemia (HL) is a marker of dysoxic metabolism, and is associated with bad outcomes in cardiac surgery. This study explores the relationship between nadir HCT on CPB and early postoperative HL.

**Design:**

Retrospective study on 3,851 consecutive patients.

**Measurements and Main Results:**

Nadir HCT on CPB and other potential confounders were explored for association with blood lactate levels at the arrival in the Intensive Care Unit (ICU), and with the presence of moderate (2.1 – 6.0 mMol/L) or severe (> 6.0 mMol/L) HL. Nadir HCT on CPB demonstrated a significant negative association with blood lactate levels at the arrival in the ICU. After adjustment for the other confounders, the nadir HCT on CPB remained independently associated with moderate (odds ratio 0.96, 95% confidence interval 0.94-0.99) and severe HL (odds ratio 0.91, 95% confidence interval 0.86-0.97). Moderate and severe HL were significantly associated with increased morbidity and mortality.

**Conclusions:**

Hemodilution on CPB is an independent determinant of HL. This association, more evident for severe HL, strengthens the hypothesis that a poor oxygen delivery on CPB with consequent organ ischemia is the mechanism leading to hemodilution-associated bad outcomes.

## Introduction

The nadir hematocrit (HCT) on cardiopulmonary bypass (CPB) is widely recognized as a risk factor for postoperative acute kidney injury [1–4], stroke [5,6], and mortality [5]. The commonly accepted interpretation for this association is that running CPB at a very low HCT value may determine a poor oxygen delivery, leading to end organ dysoxia and consequent organ failure [7–9].

A prolonged condition of poor oxygen delivery triggers the anaerobic energy production, leading to increased levels of blood lactates [10]. Hyperlactatemia (HL) during CPB is associated with a low oxygen delivery [11] and with bad outcomes in adult [12] and pediatric patients [13]. HL immediately following heart surgery is a marker of an impaired hemodynamic condition and is associated with an increased morbidity and mortality [14–16].

It is therefore reasonable to hypothesize that the nadir HCT on CPB may be a determinant of early postoperative HL through the exposure of the patient to a poor oxygen delivery during CPB, consequently linking hemodilution, inadequate oxygen delivery, and early postoperative HL to major morbidity and mortality.

Presently, there is a gap of knowledge with respect to the association of the nadir HCT on CPB with early postoperative HL. The present study aims to verify the hypothesis that low values of nadir HCT on CPB are associated with increased levels of blood lactates at the arrival in the ICU, therefore confirming the dysoxic interpretation of the link between nadir HCT on CPB and bad outcomes.

## Methods

### Study design

This is a retrospective study, based on our institutional database. The Local Ethics Committee (IRCCS San Raffaele Hospital) approved this study, waiveing the need for an informed consent from the patients. At the hospital admission, all the patients gave written approval to the treatment of their data in an anonymous form and for scientific purposes.

### Patients

We analyzed data routinely collected in our institutional database from January 1^st^, 2010, through December 31^st^, 2013. Routine inclusion of blood gas analysis data at the arrival in ICU (including blood lactate values) was available in this period. The database includes all the patients receiving a cardiac surgery operation, with the exclusion of transplant operations (not performed at our Institution). The initial patient population included 5,645 patients. Patients aged < 18 years and patients receiving an off-pump procedure were excluded from the study, reaching a study population of 3,851 patients.

### Data collection and definitions

For each patient, the following data were collected and available:

Preoperative: demographics; left ventricular ejection fraction (%); preoperative HCT (%); congestive heart failure; cardiogenic shock; active endocarditis; unstable angina; preoperative intra-aortic balloon pump; serum creatinine value (mg/dL); serum bilirubin value (mg/dL); chronic dialysis; chronic obstructive pulmonary disease; diabetes (on medication); previous cerebrovascular accident; previous cardiac surgery; non-elective procedures. Operative: type of operation (other than isolated coronary surgery; combined surgery [coronary + valve surgery or double/triple valve surgery], mitral valve surgery; aortic valve surgery; ascending aorta surgery); cardiopulmonary bypass (CPB) duration (minutes); nadir HCT on CPB; nadir temperature (°C) on CPB. Postoperative: blood lactate values (mMol/L) at the arrival in the ICU; stroke; acute kidney injury (peak postoperative serum creatinine double the baseline value); bloodstream infections (with positive cultures); operative (in-hospital or within 30 days after discharge) mortality.

For the purposes of the present analysis, patients were attributed to three groups according to the blood lactate values at the arrival in the ICU: no HL (lactate value ≤ 2.0 mMol/L), moderate HL (lactate value 2.1–6.0 mMol/L) and severe HL (lactate value > 6.0 mMol/L).

### Surgery and CPB

Patients were generally treated under moderate hypothermia (32°C—34°C) unless for specific procedures. Roller or centrifugal pumps were used, the CPB circuit was primed with colloid solutions at variable volumes, ranging from 800 to 1,200 mL. Pump flow was set between 2.0 and 2.8 L^.^ min^-1^ m^-2^, and adjusted according to the temperature and the HCT value. The heart was arrested using antegrade cold crystalloid cardioplegia or cold blood cardioplegia according to the surgeon’s preference. Anticoagulation was achieved with unfractionated heparin according to our standard protocols (loading doses 300 IU/kg to reach a target activated clotting time of 450–480 seconds; additional doses of 80 IU to maintain this value), and heparin reversal was achieved with adequate doses of protamine sulfate. All the patients received tranexamic acid at a dose of 15 mg/kg before CPB and 15 mg/kg after protamine administration. Cell-saver was used during the operation in selected cases.

### Statistics

All data are presented as number with percentage for categorical variables, mean with standard deviation for normally distributed continuous variables, and median with interquartile range for continuous, non-normally distributed variables. Normality of distribution was checked with the Kolgomorov-Smirnov test.

The association between continuous variables was tested using polynomial regression analyses, testing different equations, and the best model was identified based on the R^2^ value.

Differences between groups were tested with the Pearson’s Chi square test, the Student’s t test, and the Mann-Whitney test when appropriate.

The variables being significantly associated with moderate and severe HL were entered into two multivariable logistic regression analyses (one for moderate HL and one for severe HL), producing odds ratios with 95% confidence interval. A maximum of one variable per each 10 events was admitted to the model; however, due to the expected large number of HL events, no over-fitting of the model was anticipated. Multi-collinearity among the independent variables in multivariable logistic regression analyses was checked using collinearity statistics with measurement of condition indices and Eigenvalues. A condition index greater than 30 was considered indicative for multi-collinearity.

All tests were two-sided. A p-value < 0.05 was considered significant for all statistical tests. Statistical calculations were performed using a computerized statistical program (SPSS 13.0, Chicago, IL).

## Results

Demographics and general characteristics of the patient population are depicted in Table 1. Overall, moderate HL at the arrival in the ICU was observed in 837 (21.7%) patients and severe HL in 153 (4.0%). The values of nadir HCT on CPB were 27.4±3.9 in the no-HL group, 26.5±4.1 in the moderate HL group, and 25.4±4.3 in the severe HL group (P = 0.001 for between-groups difference).

**Table 1:** Demographics, risk profile, and serum blood lactates of the patient population (N = 3,851).

Variable	Data
Age (years)	69 (59–76)
Weight (kgs)	73.5 (64–82)
Gender male	2,584 (67.1)
Left ventricular ejection fraction (%)	55 (48–61)
Congestive heart failure	334 (8.7)
Cardiogenic shock	18 (0.5)
Preoperative intra-aortic balloon pump	60 (1.6)
Active endocarditis	86 (2.2)
Serum creatinine (mg/dL)	1.0 (0.8–1.2)
Serum bilirubin (mg/dL)	0.5 (0.4–0.7)
Chronic dialysis	32 (0.8)
Previous stroke	100 (2.6)
Diabetes on medication	702 (18.2)
Chronic obstructive pulmonary disease	281 (7.3)
Preoperative hematocrit (%)	39 (35.8–41.9)
Redo surgery	280 (7.3)
Other than isolated coronary surgery	2,574 (66.8)
Complex surgery	1,201 (31.2)
Mitral valve surgery	990 (25.7)
Aortic valve surgery	1,382 (35.9)
Ascending aorta surgery	287 (7.5)
Non-elective procedure	408 (10.6)
CPB duration (min)	76 (58–102)
Nadir hematocrit on CPB (%)	27 (24–30)
Nadir temperature on CPB (°C)	32 (32–33)
Serum blood lactates at the arrival in ICU (mmol/L)	1.4 (1.0–2.1)
Moderate hyperlactatemia	837 (21.7)
Severe hyperlactatemia	153 (4.0)

Data are number (%) or median (interquartile range).

CPB: cardiopulmonary bypass; HCT: hematocrit; ICU: intensive care unit; IQ: interquartile range.

**Fig 1:**
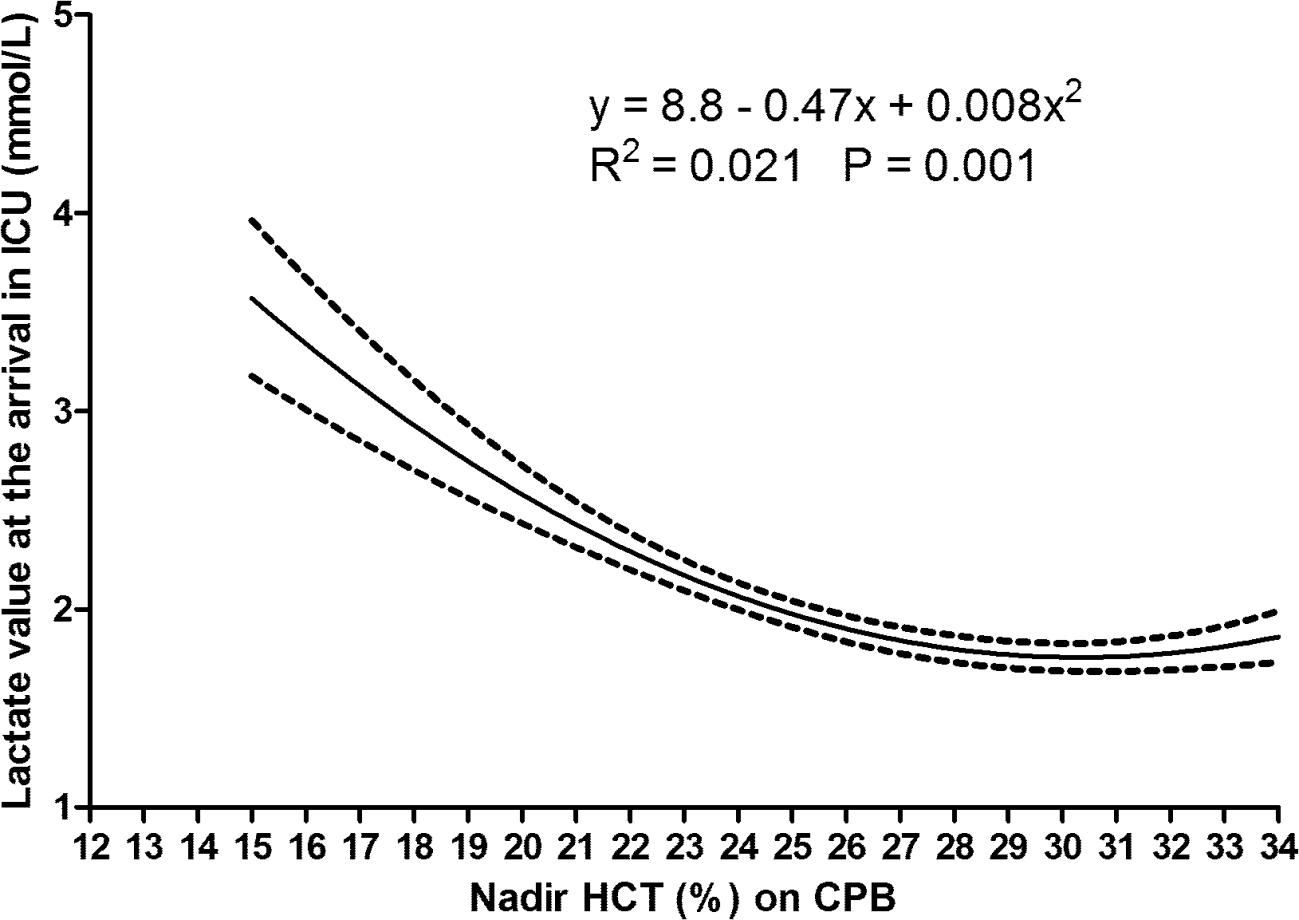
Univariate association (quadratic polynomial regression) between nadir hematocrit (HCT) on cardiopulmonary bypass (CPB) and blood lactate value at the arrival in the Intensive Care Unit (ICU). Dashed lines: 95% confidence interval.

The univariate association between nadir HCT on CPB and levels of lactates at the arrival in the ICU was defined by a quadratic equation (Fig 1) with higher values of lactates observed for lower values of nadir HCT.

Among the variables considered, 19 factors were significantly associated with moderate HL, severe HL, or both (Table 2).

**Table 2:** Univariate association between perioperative factors and postoperative hyperlactatemia.

Variable	No HL (N = 2,861)	Moderate HL (N = 837)	P	Severe HL (N = 153)	P
	57 (50–62)	50 (35–58)	0.001	40 (30–54)	0.001
Left ventricular ejection fraction (%)	185 (6.5)	124 (14.8)	0.001	25 (16.3)	0.001
Congestive heart failure	6 (0.2)	6 (0.7)	0.034	6 (3.9)	0.001
Cardiogenic shock	29 (1.0)	22 (2.6)	0.001	9 (5.9)	0.001
Preoperative intra-aortic balloon pump	45 (1.6)	32 (3.8)	0.001	9 (5.9)	0.001
Active endocarditis	0.9 (0.8–1.1)	1.0 (0.9–1.3)	0.001	1.1 (0.9–1.4)	0.001
Serum creatinine (mg/dL)	17 (0.6)	15 (1.8)	0.002	0 (0)	0.339
Chronic dialysis	59 (2.1)	36 (4.3)	0.001	5 (3.3)	0.313
Previous stroke	194 (6.8)	77 (9.2)	0.018	10 (6.5)	0.901
Chronic obstructive pulmonary disease	39 (36–42)	38.5 (35–42)	0.001	37.4 (33–41)	0.001
Preoperative hematocrit (%)	168 (5.9)	88 (10.5)	0.001	24 (15.7)	0.001
Redo surgery	1,855	598 (71.4)	0.001	121 (79.1)	0.001
Other than isolated coronary surgery	758 (26.5)	368 (44.0)	0.001	75 (49.0)	0.001
Complex surgery	667 (23.3)	272 (32.5)	0.001	51 (33.3)	0.005
Mitral valve surgery	1,071 (37.4)	262 (31.3)	0.001	49 (32.0)	0.171
Aortic valve surgery	230 (8.0)	127 (15.2)	0.001	51 (33.3)	0.001
Non-elective procedure	73 (56–95)	88 (64–123)	0.001	122 (86–185)	0.001
CPB duration (min)	27 (25–30)	26 (24–29)	0.001	25 (22–28)	0.001
Nadir hematocrit on CPB (%)	32.2 (32.2–33)	32 (31.3–33)	0.001	32 (30–32.6)	0.001
Nadir temperature on CPB (°C)	57 (50–62)	50 (35–58)	0.001	40 (30–54)	0.001

Data are median (IQ range) or number (%).

CPB: cardiopulmonary bypass; HCT: hematocrit; HL: hyperlactatemia; IQ: interquartile

Factors being associated with moderate or severe HL at the univariate analysis were entered into two distinct multivariable logistic regression analyses having moderate or severe HL as dependent variables. CPB duration was not included in the model, due to severe multi-collinearity with the other independent variables (ejection fraction, serum creatinine, congestive heart failure, preoperative IABP, active endocarditis, redo surgery, non-elective surgery, non-isolated coronary surgery, nadir temperature on CPB, and nadir HCT on CPB) with a condition index of 84.9.

After correction for the potential confounders (Table 3), the nadir HCT on CPB remained an independent risk factor for both moderate and severe HL. The relative risk of HL increased by 4% per each unit decrease of the nadir HCT on CPB for moderate HL, and by 9% for severe HL.

**Table 3:** Multivariable logistic regression analyses for moderate and severe postoperative hyperlactatemia.

Variable	Moderate hyperlactatemia			Severe hyperlactatemia		
	B coefficient	O.R. (95% C.I.)	P	B coefficient	O.R. (95% C.I.)	P
Left ventricular ejection fraction (%)	- 0.060	0.94 (0.93–0.95)	0.001	- 0.087	0.92 (0.90–0.93)	0.001
Congestive heart failure	0.402	1.49 (1.13–1.97)	0.004	Not independently associated		
Serum creatinine (mg/dL)	0.161	1.17 (1.02–1.36)	0.029	Not independently associated		
Previous stroke	0.483	1.62 (1.01–2.60)	0.045	Not independently associated		
Preoperative hematocrit	- 0.023	1.02 (1.00–1.04)	0.039	Not independently associated		
Redo surgery	0.545	1.72 (1.27–2.34)	0.001	1.047	2.85 (1.63–5.00)	0.001
Complex surgery	0.467	1.59 (1.29–1.98)	0.001	0.479	1.61 (1.01–2.58)	0.044
Aortic valve surgery	- 0.395	0.67 (0.53–0.86)	0.001	Not independently associated		
Non-elective procedure	0.324	1.38 (1.05–1.81)	0.020	1.298	3.66 (2.31–5.81)	0.001
Nadir hematocrit on CPB (%)	- 0.035	0.96 (0.94–0.99)	0.009	- 0.094	0.91 (0.86–0.97)	0.002
Nadir temperature on CPB (°C)	- 0.140	0.87 (0.82–0.92)	0.001	- 0.193	0.83 (0.75–0.90)	0.001
Constant	6.902			8.042		

B: regression; CABG: coronary artery bypass graft; CPB: cardiopulmonary bypass; C.I.: confidence interval; HL: hyperlactatemia; O.R.: odds ratio.

**Fig 2:**
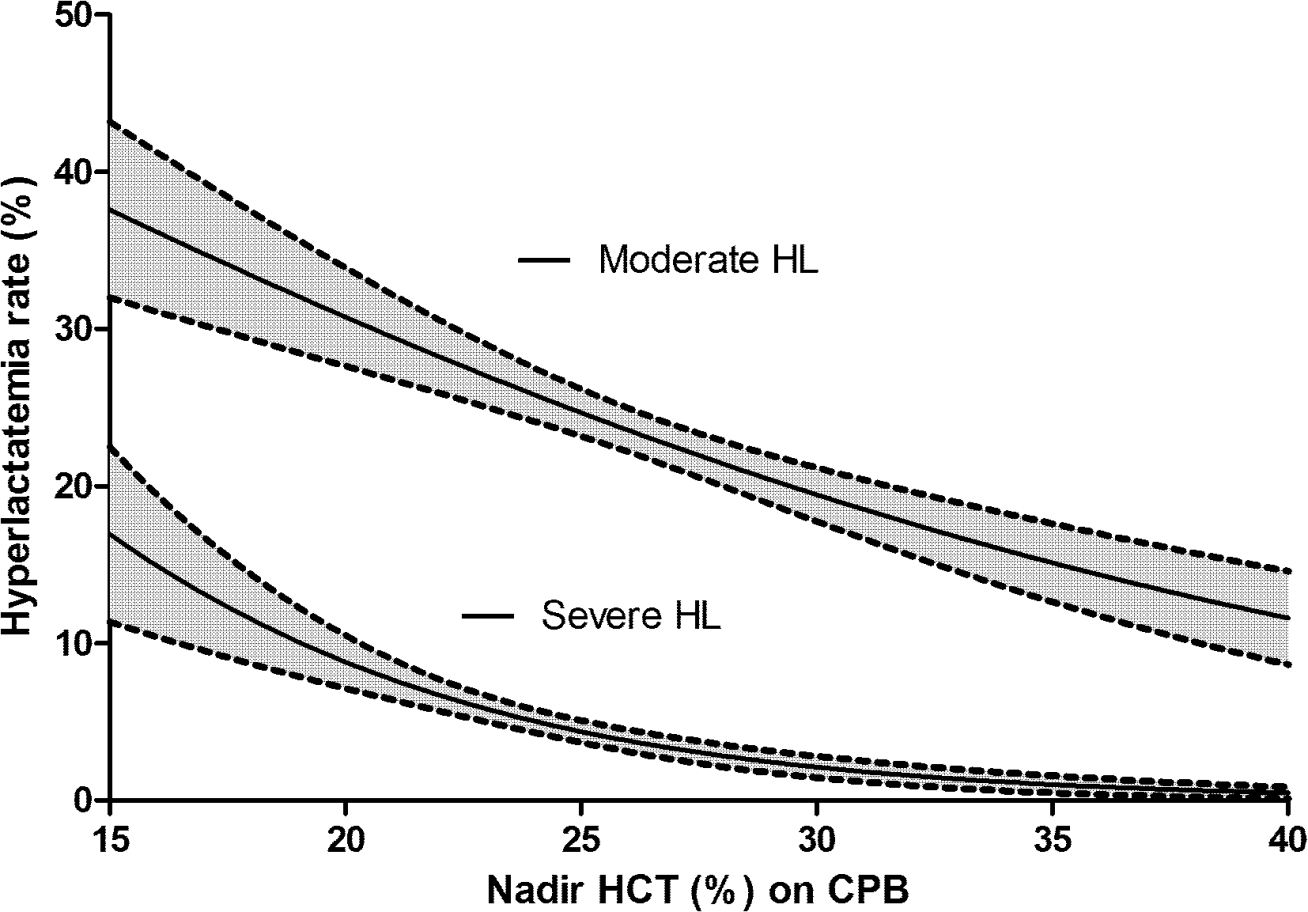
Univariate association (logistic regression) between nadir hematocrit (HCT) on cardiopulmonary bypass (CPB) and moderate or severe hyperlactatemia (HL) rate at the arrival in the Intensive Care Unit. Dashed lines: 95% confidence interval.

The unadjusted relationship between nadir HCT on CPB and moderate or severe HL is defined by logistic regressions shown in Fig 2. Within the considered range of nadir HCT values, the relationship is almost linear for moderate HL, with an absolute risk of moderate HL of 25% at a nadir HCT on CPB of 25%, increasing to 31% for a nadir HCT value of 20% (relative risk increase 20%). Conversely, the absolute risk of severe HL is 4.3% at a nadir HCT on CPB of 25%, increasing to 8.7% at a nadir HCT on CPB of 20% (relative risk increase 100%).

The clinical outcome of the patients according to the presence of moderate or severe HL is shown in Table 4. Major morbidity and operative mortality were significantly worse in both moderate and severe HL groups.

**Table 4:** Clinical outcome in presence of no hyperlactatemia, moderate or severe hyperlactatemia.

Variable	No HL (N = 2,861)	Moderate HL (N = 837)	P	Severe HL (N = 153)	P
Acute kidney injury	56 (2.0)	40 (4.8)	0.001	21 (13.7)	0.001
Stroke	3 (0.1)	7 (0.8)	0.001	4 (2.6)	0.001
Bloodstream infections	118 (4.1)	84 (10)	0.001	28 (18.3)	0.001
Operative mortality	41 (1.4)	60 (7.2)	0.001	32 (20.9)	0.001

Data are number (%).

HL: hyperlactatemia

## Discussion

This study demonstrates, in a large series of patients who underwent cardiac surgery with CPB, that the nadir HCT on CPB is independently associated with both moderate and severe HL. The impact of the nadir HCT on CPB as a risk factor for HL is more pronounced for severe HL: the same decrease of nadir HCT on CPB induces an increase in the relative risk of HL that is 4 times higher for severe HL than for moderate HL.

Additionally, this study is confirmative of the previous findings [12–16] linking postoperative HL with major morbidity and mortality.

HL is the consequence of lactic acidosis, through the buffering of lactic acid by anions. There are two different species of L-lactic acidosis. Type A recognizes a hypoxic nature, is a marker of shock, and is found during septic shock, mesenteric ischemia, hypoxemia, hypovolemic or cardiogenic shock, poisoning by carbon monoxide or cyanide [10]. Type B is non-hypoxic and may be related to medications, thiamine deficiency, and intoxications. In the setting of cardiac surgery, type A is the dominant pattern [14–18]. However, the temporal aspects of postoperative HL are important to correctly interpret this finding. HL during CPB is generally associated with the exposure to an inadequate oxygen delivery, in particular during the rewarming phase [12,13]. HL at the arrival in the ICU (otherwise defined “early HL”) may reflect an inadequate oxygen delivery during CPB or after weaning from CPB, or both. It is difficult or even impossible, with an isolated blood lactate measure, to draw conclusions about the exact onset of HL, because in presence of an organ dysoxia blood lactate formation is rapidly triggered; however, lactate clearance takes more time, is a liver-dependent phenomenon, and may be hampered itself by a condition of low hepatic blood flow [19]. In our study, we measured blood lactate at the arrival in the ICU and we addressed moderate and severe early HL. It is likely that some patients may have started lactate formation during CPB, while others may not. However, the finding that the nadir HCT on CPB is independently associated especially with severe HD is suggestive for a hemodilution-related (low oxygen content) type A HL at least in a percentage of our patients. Conversely, other patients may have faced a condition of low cardiac output only once weaned from CPB, leading to a different kind of type A HL (low oxygen transport).

There are few studies linking oxygen delivery on CPB with early postoperative HL. However, Demers and associates [12] could notice that low levels of hemoglobin on CPB were associated with the onset of HL during CPB. More importantly, Abraham and associates [20] observed that postoperative HL in children undergoing atrial septal repair was associated with lower rates of pump flow and oxygen delivery.

The notion that severe hemodilution on CPB may be deleterious, and that every effort should be applied to avoid it, is nowadays generally recognized. However it is still unclear whether the relationship between the nadir HCT on CPB and bad outcomes is directly causative or rather an epiphenomenon, a reflection of other pathological conditions (like preoperative anemia), or even simply the trigger for allogeneic blood product transfusions-related adverse effects. The present study suggests, in a large series of patients, that even after correction for potential confounders hemodilution on CPB accounts for a certain percentage of patients experiencing moderate or severe early postoperative HL. The dysoxic chain represented by hemodilution / poor oxygen delivery / organ ischemia / hyperlactatemia / bad outcomes could explain many of the complications observed in presence of very low values of HCT on CPB.

This study has limitations. The most important is the absence of additional available data during CPB, like the pump flow and the oxygen delivery. This would have been useful to better elucidate the link between oxygen delivery and postoperative HL. As a matter of fact, it is our routine policy is to adjust the pump flow according to the HCT in order to maintain an adequate oxygen delivery. Our data suggest that, despite this strategy, very low levels of HCT are determinants of anaerobic production of lactates. A second potentially useful lacking datum is the time of exposure to the nadir HCT while on CPB. If the hemodilution is leading to a poor oxygen delivery, then the longer is the exposure, the more critical will be the organ ischemia and the higher will be the value of postoperative lactates. Further studies are certainly warranted to elucidate the role of time within the context of the relationship between hemodilution, HL, and outcome.

In conclusion, our study confirms the role of early postoperative HL as a predictive marker of bad outcomes in cardiac surgery, and highlights the role of hemodilution on CPB as an independent determinant of moderate and severe early postoperative HL. Hemodilution on CPB is far from being the only or the major factor leading to postoperative HL: other mechanisms, occurring before, during, and after CPB may be advocated as determinants of type A postoperative HL. However, in order to limit the modifiable mechanism linking hemodilution, HL, and bad outcomes, possible interventions include (i) the prevention of severe hemodilution by reduction of the CPB circuit priming volume and (ii) the increase of pump flow to compensate severe hemodilution and guarantee an adequate oxygen delivery. Further prospective studies exploring these strategies are needed.
